# Anaesthetics modulate tumour necrosis factor α: effects of L-carnitine supplementation in surgical patients. Preliminary results.

**DOI:** 10.1155/S0962935193000730

**Published:** 1993

**Authors:** Giovanna Delogu, Claudio De Simone, Giuseppe Famularo, Alessandra Fegiz, Francesca Paoletti, Emilio Jirillo

**Affiliations:** 1 Anaesthesiology and Intensive Care University “La Sapienza” Rome Italy; 2 Infectious Diseases University of L'Aquila Italy; 3 Internal Medicine University of L'Aquila Italy; 4 Infectious Diseases University “La Sapienza” Rome Italy; 5 Immunology University of Bari Italy

## Abstract

Both anaesthetics and surgical trauma could strongly affect the production of tumour necrosis factor α (TNFα). During *in vitro* experiments the authors found that anaesthetics modulate the production of TNFα by peripheral blood mononuclear cells. Notably, Pentothal strongly increased the production of the cytokine as compared to both lipopolysacchride treated and control mononuclear cells, whereas in supernatants from Leptofen driven mononuclear cells TNFα was strongly reduced. On the other hand, Pavulon did not significantly affect the cytokine production. In the *in vivo* study, in an attempt to ameliorate the metabolic response to surgical trauma, L-carnitine was administered to 20 surgical patients, then the circulating TNFα was measured. The results indicate that the levels of circulating TNFα were strongly increased following surgery and that L-carnitine administration resulted in a strong reduction of TNFα. Thus, the data suggest that L-carnitine could be helpful in protecting surgical patients against dysmetabolism dependent on dysregulated production of TNFα.


Research Paper

Mediators of Inflammation 2, S33–S36 (1993)

### Anaesthetics modulate tumour necrosis factor α: effects of L-carnitine supplementation in surgical patients. Preliminary results.

Giovanna Delogu,[1] Claudio De Simone,[2,CA] Giuseppe Famularo,[3] Alessandra Fegiz,[1] Francesca Paoletti[4] and Emilio Jirillo[5]

[1]Anaesthesiology and Intensive Care and [4]Infectious Diseases, University "La Sapienzie" Rome; [2]Infectious Diseases and [3]Internal Medicine, University of L'Aquila; [5]Immunology, University of Bari, Italy.

[CA] Corresponding Author

**BOTH anaesthetics and surgical trauma could strongly affect the production of tumour necrosis factor α (TNFα). During *in vitro* experiments the authors found that anaesthetics modulate the production of TNFα by peripheral blood mononuclear cells. Notably, Pentothal strongly increased the production of the cytokine as compared to both lipopolysacchride treated and control mononuclear cells, whereas in supernatants from Leptofen driven mononuclear cells TNFα was strongly reduced. On the other hand, Pavulon did not significantly affect the cytokine production. In the *in vivo* study, in an attempt to ameliorate the metabolic response to surgical trauma, L-carnitine was administered to 20 surgical patients, then the circulating TNFα was measured. The results indicate that the levels of circulating TNFα were strongly increased following surgery and that L-carnitine administration resulted in a strong reduction of TNFα. Thus, the data suggest that L-carnitine could be helpful in protecting surgical patients against dysmetabolism dependent on dysregulated production of TNFα.**

**Key words:** Anaesthetics, L-Carnitine, Surgery, TNFα

### Introduction

Patients undergoing surgery may subsequently have life-threatening multiple organ failure (MOF).[1] The dysregulated release of cytokines by macrophages is likely to play a major role in the pathogenesis of this disorder.[2] Among macrophage derived cytokines, TNFα is pivotal since it is directly involved in mediating the organism adaptative response to injury, including surgery.[3,4]

Several anaesthetic agents are able to affect the pattern of cytokine production by monocytes/macrophages *in vitro*, including TNFα, and several reports have confirmed that the confirmed administration of anaesthetics, profoundly derange the normal functions of the immune system *in vivo*.[5–9]

Here, the results of experiments are reported, which indicate that anaesthetic drugs, most notably Pentothal, could strongly enhance under *in vitro* conditions the TNFα production by peripheral blood mononuclear cells (PBMCs) from healthy individuals as compared to both lipopolysaccharide (LPS) treated and control cells. In addition, in *in vivo* studies the authors found that serum levels of TNFα are significantly increased following surgery. The administration of L-carnitine resulted in the reduction of circulating TNFα.

### Materials and Methods

In vitro *experiments*:

*PBMCs.* PBMCs were obtained from healthy volunteers by standard methods, as described previously.[10]

*Cell cultures.* Briefly, PBMCs were resuspended at the concentration of $2 \times 10^6$ cells/ml in RPMI 1640 (Gibco Bio Cult., Paisley, UK) containing 10% heat-inactivated foetal calf serum, 1% L-glutamine, and penicillin/streptomycin. PBMCs were cultured for 72 h in a 5% $CO_2$ atmosphere at 37°C in the presence of either standard LPS concentration, as described previously[10] or anaesthetic drugs (Leptofen, Pavulon, Pentothal) at the optimal concentration of 10 μg/ml. At the end of the culture period, the supernatants were harvested and TNFα was measured.

*Measurement of TNFα.* TNFα was quantified by a sandwich enzyme immunoassay (Biokine, T Cell Sciences Inc., MA, USA), according to standard methods.[11] The lower limit of TNFα detection in this immunoassay was 10 pg/ml. Serum TNFα measured in healthy volunteers was below this limit.

In vivo *studies:*

*Patients.* Twenty patients were admitted for surgery and randomly assigned to receive either

© 1993 Rapid Communications of Oxford Ltd

NLA anaesthesia alone (Group 1) or NLA anaesthesia plus L-carnitine (Carnitene$^{TM}$, 1 g ampoules, Sigma Tau, Pomezia, Italy) (Group 2).

No patient was suffering from cancer or infections or had received any medical treatment before the admission to the study. Surgery was always performed by the same surgeon.

The patients were well matched with respect to age (45 ± 13 and 47 ± 9 years), weight (71 ± 2 and 72 ± 8 kg), and height (166 ± 24 and 170 ± 26 cm), in Group 1 and Group 2, respectively. Surgery was performed because of nephrolithiasis (one and two patients), uterocele (one and zero), hysteromyoma (one and one), colonic diverticula (four and five), megacolon (two and two), and irritable bowel syndrome (one and zero) in Group 1 and Group 2, respectively. The type of surgery was colonic resection in three and two, nephrectomy in one and two, hysterectomy in two and one, and hemicolectomy in four and five patients, in Group 1 and Group 2, respectively. Notably, the duration of surgery was comparable in both groups (123 ± 61 and 139 ± 52 min in Group 1 and Group 2, respectively) and the post-surgery course was not burdened by complications.

Informed consent was obtained from all subjects and the study was approved by the Hospital Ethics Committee on Human Research.

*Anaesthetic technique.* Premedication was performed with atropine sulphate (0.5 mg i.m.) and diazepam (10 mg i.m.) administered 40–50 min before the scheduled time of surgery. Then, anaesthesia was induced with droperidol (2.5 mg), fentanyl (0.1 mg) and thiopentone (3.5 mg/kg). Tracheal intubation was performed after administering pancuronium (0.08 mg/kg). Anaesthesia was maintained with 60% nitrous oxide in oxygen. Supplementary doses of fentanyl, droperidol, and pancuronium were given as needed. Mean doses were fentanyl, 0.056 ± 0.02 mg; pancuronium, 8.3 ± 0.6 mg; droperidol, 3.8 ± 0.5 mg. At the end of surgery, residual neuromuscular blockade was antagonized with a mixture of atropine and neostigmine. All anaesthetic drugs were administered by the same anaesthetist.

*Post-surgery care.* All patients in both groups were given buprenorphine 0.3 mg for analgesia on the day of surgery only.

*L-carnitine treatment.* In Group 2 patients, L-carnitine was administered i.v. at the dosage of 8 g at the end of surgery and 24 h afterwards.

*Blood sampling.* Samples of peripheral blood were obtained for TNFα measurement on the day before surgery and 2, 24, and 48 h after surgery (t0, t1, t2, t3, respectively).

Samples of peripheral blood were obtained for carnitine measurement at t0, t2, and t3, respectively. Since the time lag between the end of surgery (L-carnitine administration) and blood drawing (2 h) was too short, no samples were collected at t1. In Group 2 patients, samples of peripheral blood were obtained before L-carnitine administration.

*L-carnitine measurement.* Serum levels of carnitines were measured by using standard methods, as described previously.[12] Briefly, a neutralized perchloric acid extract of plasma was incubated with $^{14}$C-acetyl coenzyme A (CoA) in the presence of carnitine acetyltransferase (CAT). Then, the labelled acetyl L-carnitine was separated from the unreacted $^{14}$C-acetyl CoA by an anion exchange resin and radioactivity counts were measured in the supernatant. This method allows the quantitative evaluation of L-carnitine (free carnitine) and L-carnitine formed from alkaline hydrolysis as an expression of the total acid soluble L-carnitine. Short-chain L-carnitine esters were measured as the difference between total acid soluble L-carnitine and free L-carnitine.

*Statistical analysis:* Results are expressed as the mean ± standard deviation. The differences between groups for paired and unpaired data were statistically significant for $p$ less than 0.01.

### Results

In vitro *experiments:* The LPS stimulation of PBMCs proved to strongly increase the TNFα release in culture media as compared to unstimulated PBMCs ($p < 0.05$), as expected (Fig. 1). However, Pentothal was significantly more effective with respect to LPS in inducing TNFα synthesis and release by PBMCs ($p < 0.001$) (Fig. 1). The stimulation of PBMCs with other anaesthetic drugs, such as Pavulon and Leptofen, did not result in increased TNFα production. In supernatants from Leptofen driven PBMC cultures TNFα levels were strongly reduced compared to both LPS driven and unstimulated cultures ($p < 0.05$), whereas in supernatants from Pavulon driven PBMC cultures TNFα was similar to that in unstimulated cultures (Fig. 1).

In preliminary experiments, the addition of L-carnitine at various doses to anaesthetic driven PBMC cultures was followed by conflicting effects on the pattern of TNFα production (data not shown). Further studies to establish both the optimal dosage and kinetics of adding L-carnitine to PBMC cultures in order to obtain a clear-cut modulation of anaesthetic driven TNFα production are therefore needed.

In vivo *studies*: Patients in Groups 1 and 2 were well matched for age, weight, height, types and/or

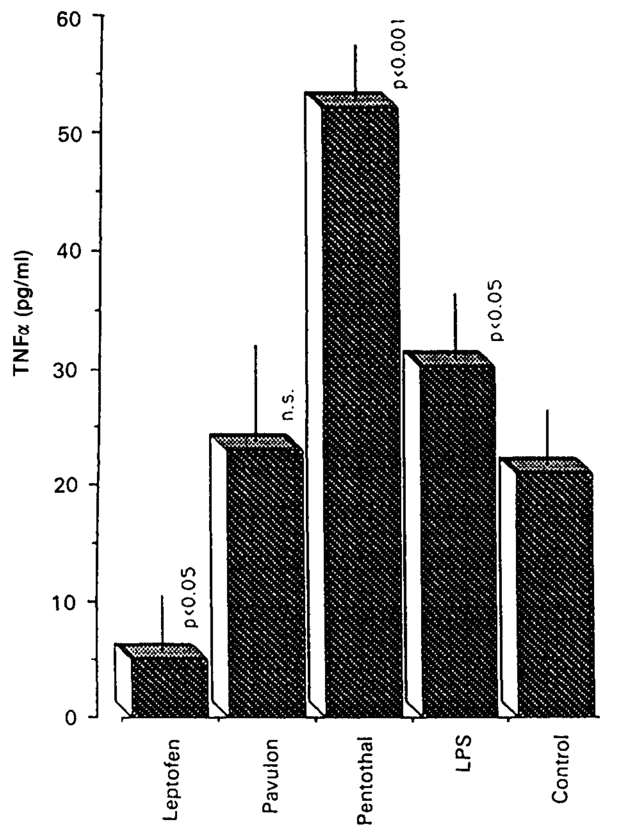

FIG. 1. TNFα levels in supernatants of PBMCs driven by either anaesthetics (Leptofen, Pavulon, Pentothal) or LPS as well as in supernatants of unstimulated PBMCs. Anaesthetics were used at the optimal concentration of 10 μg/ml. The results are shown as the mean levels of TNFα of triplicate experiments ± standard deviation. See Materials and Methods for details.

duration of surgery. During surgery, blood loss ranged from 600 to 1 200 ml. Nevertheless, patients did not require blood transfusion and received conventional crystalloid or colloid (Dextran 70) fluids. Both Group 1 and Group 2 patients did not exhibit any complication throughout their course and the body temperature following surgery did not exceed 38.2°C. The pattern was similar in both groups. Finally, any treatment related adverse effect was recorded in patients receiving L-carnitine.

The effects of surgical/anaesthetic trauma on TNFα serum levels are shown in Table 1. At t0, all patients had circulating TNFα within the normal range. In Group 1, a significant increase of plasma levels of TNFα was found throughout t1 (40 ± 2 pg/ml; $p < 0.01$) to t3 (62 ± 7 pg/ml; $p < 0.001$), peaking at t2 (68 ± 8 pg/ml; $p < 0.001$), with respect to serum levels at t0 (5 ± 2 pg/ml). In Group 2 patients strongly increased ($p < 0.01$) serum levels of TNFα were found only at t1 (30.3 ± 5 pg/ml) compared to t0 (6 ± 2 pg/ml); at t2 (15 ± 4 pg/ml) and t3 (22 ± 9 pg/ml) TNFα was not significantly different with respect to t0. Notably, serum levels of TNFα were significantly different between Group 1 and Group 2 patients only at t2 ($p < 0.001$) and t3 ($p < 0.01$).

Total, free, and short-chain carnitine levels were comparable in the two groups and within the normal range at t0 (Table 2). In Group 1 patients, no significant change of total, free, and short-chain carnitine was found at t2 and t3 (Table 2). However, in Group 2 patients serum carnitine levels were increased significantly at t2 and t3 with respect to baseline values, as expected (Table 2).

**Table 1.** Serum levels of TNFα (pg/ml) following surgical/anaesthetic trauma in patients receiving placebo (Group 1) and L-carnitine (Group 2)

| Time | Patients | |
|---|---|---|
| | Group 1 | Group 2 |
| t0 | 5 ± 2 | 6 ± 2 |
| t1 | $40 \pm 2^{*}$ | $30 \pm 5^{*}$ |
| t2 | $68 \pm 8^{**a}$ | 15 ± 4 |
| t3 | $62 \pm 7^{**b}$ | 22 ± 9 |

$^{*}p < 0.01$ with respect to t0; $^{**}p < 0.001$ with respect to t0; $^{a}p < 0.001$ with respect to Group 2; $^{b}p < 0.01$ with respect to group 2. For t0, t1, t2, and t3 see Materials and Methods.

### Discussion

The results indicate that anaesthetic drugs can affect TNFα production and release by monocytes/macrophages, under *in vitro* conditions. However, strong differences exist among anaesthetic drugs in their ability to modulate TNFα production, as suggested by the finding that Pentothal strongly enhanced the cytokine production compared to LPS, whereas both Pavulon and Leptofen did not. The demonstration that Pentothal is effective in inducing TNFα is consistent with the hypothesis that surgical patients treated with Pentothal could be at risk of MOF, since TNFα is

**Table 2.** Total, free, and short-chain carnitine (nmol/ml) in serum from patients receiving placebo (Group 1) and L-carnitine (Group 2)

| Time | Patients | |
|---|---|---|
| | Group 1 | Group 2 |
| Total carnitine | | |
| t0 | 30 ± 7 | 34 ± 8 |
| t2 | 50 ± 9 | $312 \pm 71^{*}$ |
| t3 | 39 ± 4 | $566 \pm 215^{*}$ |
| Free carnitine | | |
| t0 | 34 ± 7 | 28 ± 8 |
| t2 | 39 ± 6 | $291 \pm 72^{*}$ |
| t3 | 28 ± 8 | $518 \pm 245^{*}$ |
| Short-chain carnitine | | |
| t0 | 6.5 ± 3.5 | 9.7 ± 4.8 |
| t2 | 5.1 ± 1.9 | $19 \pm 6^{*}$ |
| t3 | 4.5 ± 2.7 | $34 \pm 5^{*}$ |

$^{*}p < 0.001$ with respect to both t0 and Group 1. For t0, t2, and t3. See Materials and Methods.

directly involved in the pathogenesis of the disorder. However, anaesthetic drugs *per se* could also down-modulate the production of TNFα, as shown by Leptofen, or not exhibit any effect at all, as is the case of Pavulon. Therefore, the pathophysiological pathways leading to the increased serum levels of TNFα following surgery, which were demonstrated in the *in vivo* study, may not be strictly dependent on the immunomodulating properties of the anaesthetic drugs *per se*. Further studies are needed to fully understand the complex network of immune and endocrine events which account for the increased TNFα production following surgical injury. In fact, the recently reported ability of benzodiazepines to modulate IL-1, IL-6, and TNFα synthesis by human monocytes/macrophages[13] further emphasizes the complexity of the pathophysiological phenomena occurring throughout anaesthesia and surgery.

In the patients enrolled in this trial, serum carnitine levels did not substantially change following surgery, whereas increased levels were found in patients receiving L-carnitine, as expected. In these latter patients, serum TNFα was strongly reduced at t2 and t3 compared to placebo treated subjects. These preliminary data clearly suggest that the treatment with L-carnitine could protect surgical patients against the dysregulated production of monocyte/macrophage derived cytokines, most notably TNFα, which can lead to MOF and death.

### References

1. Weissman C. The metabolic response to stress: an overview and update. *Anaesthesiology* 1990; **73**: 298–327.
2. Salo M. Effects of anaesthesia and surgery on the immune response. *Acta Anaesthesiol Scand* 1992; **36**: 201–220.
3. Spooner CE. The role of tumour necrosis factor in sepsis. *Clin Immunol Immunopathol* 1992; **62**: S11–S17.
4. Tracey KJ. The acute effects of chronic pathophysiologic effects of TNF: mediation of septic shock and wasting (cachexia). In: Beutler B, ed. *Tumour necrosis factors. The molecules and their emerging role in medicine.* New York: Raven Press, 1992; 255–273.
5. Rossano F, Tufano R, Cipollone G, *et al.* Anaesthetic agents induce circulating mononuclear leukocytes to release cytokines. *Immunopharmacol Immunotoxicol* 1992; **14**: 439–450.
6. Mondgill GC. Update on anaesthesia and the immune response. *Can Anaesth Soc J* 1986; **33**: S54–S71.
7. Stevenson GW, Hall S, Rudnick SJ, *et al.* Halothane anaesthesia decreases human monocyte hydrogen peroxide generation. Protection of monocyte by activation with gamma-interferon. *Immunopharmacol Immunotoxicol* 1987; **9**: 489–494.
8. Tonnesen E, Srinkla MM, Christensen NJ, Olsen AS, Hadsen J. Natural killer activity and lymphocyte function during and after coronary artery by-pass grafting to the endocrine stress response. *Anaesthesiology* 1987; **67**: 526–530.
9. Pertilla S, Salo M, Rajanaki A. Granulocyte microbicidal function in patients undergoing major abdominal surgery under balanced anaesthesia. *Acta Anaesthesiol Scand* 1987; **31**: 100–103.
10. Famularo G, Giacomelli R, Di Giovanni S, Sacchetti S, Tonietti G. Cytokine production in patients with monoclonal gammopathies. *J Clin Lab Immunol* 1991; **34**: 63–70.
11. De Simone C, Tzantzoglou S, Famularo G, *et al.* High-dose L-carnitine improves immunologic and metabolic parameters in AIDS patients. *Immunopharmacol Immunotoxicol* 1993; (in press).
12. De Simone C, Tzantzoglou S, Jirillo E, Marzo A, Vullo V, Arrigoni Martelli E. L-carnitine deficiency in AIDS patients. *AIDS* 1992; **6**: 203–205.
13. Taupin V, Jayais P, Descamps-Latscha B, *et al.* Benzodiazepine anaesthesia in humans modulates the interleukin-1$\beta$, tumour necrosis factor alpha, and interleukin-6 responses of blood monocytes. *J Neuroimmunol* 1991; **35**: 13–19.
